# Effect of Denosumab on Glucose Homeostasis in Postmenopausal Women with Breast Cancer Treated with Aromatase Inhibitors: A Pilot Study

**DOI:** 10.1155/2020/1809150

**Published:** 2020-11-06

**Authors:** Alessandro Rossini, Sofia Frigerio, Elena Dozio, Roberto Trevisan, Gianluca Perseghin, Sabrina Corbetta

**Affiliations:** ^1^Endocrinology and Diabetes Unit, ASST Papa Giovanni XXIII, Bergamo 24127, Italy; ^2^Endocrinology Unit, Fondazione IRCCS Ca' Granda, Ospedale Maggiore Policlinico, Milan 20122, Italy; ^3^Department of Clinical Sciences and Community Health, University of Milan, Milan 20122, Italy; ^4^Department of Biomedical Sciences for Health, University of Milan, Milan 20122, Italy; ^5^Department of Medicine and Surgery, Università Degli Studi di Milano-Bicocca, Monza 20900, Italy; ^6^Department of Medicine and Rehabilitation, Policlinico Monza, Monza 20900, Italy; ^7^Endocrinology and Diabetology Service, IRCCS Istituto Ortopedico Galeazzi, Milan 20161, Italy; ^8^Department of Biomedical, Surgical and Dental Sciences, University of Milan, Milan 20122, Italy

## Abstract

**Background:**

Aromatase inhibitors in women with breast cancer have been associated with cancer treatment-induced bone loss (CTIBL), increased fracture risk, and impairment of glucose metabolism. Denosumab (Dmab), a monoclonal antibody against RANKL, which is a key regulator of the osteoclast activity, is effective as an antiresorptive agent in the treatment of CTIBL. Since RANKL/RANK pathway may contribute to the pathogenesis of glucometabolic disorders, it has been suggested that Dmab may improve glucose homeostasis. Our pilot study evaluated the effect of a single administration of 60 mg Dmab on glucose metabolism in a cohort of women with breast cancer treated with aromatase inhibitors.

**Methods:**

Fifteen postmenopausal nondiabetic women were prospectively enrolled. Oral glucose tolerance test (OGTT) and metabolic parameters, including FGF21, were assessed at baseline and one month after Dmab injection. Midterm glucose control was evaluated by measuring glycated haemoglobin (HbA1c) levels 5 months after Dmab.

**Results:**

Parameters of glucose metabolism were not different one month after Dmab but circulating FGF21 levels significantly decreased (128.5 ± 46.8 versus 100.2 ± 48.8 pg/mL; *p*=0.016). Considering patients with insulin resistance at baseline (HOMA-IR > 2.5 and Matsuda Index < 2.5; *n* = 5), reduced mean fasting insulin levels (16.3 ± 4.9 versus 13.5 ± 3.5 mcU/mL; *p*=0.029) and increased insulin sensitivity index QUICKI (0.317 ± 0.013 versus 0.327 ± 0.009; *p*=0.025) were found. Nonetheless, HbA1c increased 5 months after Dmab (36.0 ± 2.3 versus 39.6 ± 3.1 mmol/mol; *p*=0.01).

**Conclusions:**

Although RANKL blockade induced a short-term positive effect on insulin sensitivity, particularly in insulin-resistant patients, a benefit on long-term glucose metabolism was not evident. In conclusion, Dmab is safe for glucose metabolism in aromatase inhibitor-treated women with breast cancer.

## 1. Introduction

Breast cancer is the leading neoplasia in females, causing about 15% of tumor death among women [1]. Evolving therapeutic strategies led to a significant improvement in patients' survival rates [2]. In particular, aromatase inhibitors (AIs), which lower circulating estrogens limiting their proliferative effect on breast cancer cells, are now the gold standard for the hormonal therapy of postmenopausal women with breast cancer [3]. As the survival of patients improved, competing causes of mortality became more relevant [4, 5]. Women with breast cancer are characterized by higher cardiovascular mortality, due to direct cardiotoxicity of cancer treatments [6] and coexistence of classical risk factors for cardiovascular diseases [5]. The link between breast cancer and metabolic abnormalities is of clinical relevance: insulin-resistance, dyslipidemia, and abdominal fat accumulation contribute to the development of cancer through multiple mechanisms [7, 8]. On the other hand, breast cancer leads to decreased physical activity and weight gain, increasing the risk of metabolic syndrome and type 2 diabetes mellitus (T2DM) [4, 9, 10]. Furthermore, treatments against cancer worsen the metabolic abnormalities [5, 11, 12]: in particular, AIs have been associated with alterations of lipid profile [12], increased body fat, and insulin-resistance [13], though correlation with increased risk of T2DM is still controversial [14, 15].

The RANK/RANKL/osteoprotegerin (OPG) pathway is a key regulator of bone remodeling [16]. Binding of RANK to its cognate receptor RANKL stimulates osteoclast activity, increasing bone resorption [16]. Elevated RANKL activity has been demonstrated in various conditions of reduced bone mass [17]. In patients treated with AIs, decline in estrogen levels induces RANKL expression in bone cells [18] and determines an imbalance in remodeling processes leading to bone loss [19]. Denosumab (Dmab), a fully humanized monoclonal antibody directed against RANKL, inhibits the RANK/RANKL pathway, decreasing bone resorption [20]. Dmab is currently approved as an antiresorptive treatment for postmenopausal osteoporosis, and it showed efficacy also in breast cancer AI-treated women [21, 22]; it is thus currently recommended for the prevention of cancer treatment-induced bone loss (CTIBL) in these patients [23].

The RANKL/RANK pathway has been involved in the pathogenesis of metabolic syndrome. Circulating soluble RANKL levels have been associated with insulin resistance and with the number of metabolic syndrome components clustering in an individual [24]; elevated RANKL levels may confer a higher risk of developing T2DM [24]. RANK and RANKL are expressed in liver and pancreatic *β* cells [25], and binding of RANKL to RANK leads to the activation of nuclear factor-kB (NF-kB), which in turn contributes to hepatic insulin resistance and *ß* cell apoptosis [24]. In murine models, downregulation of RANKL signaling in liver tissue markedly reduced hepatic insulin resistance and ameliorated glucose metabolism [24]. Conversely, the metabolic effect of RANKL blockade in humans is still a matter of debate. Both Passeri et al. [26] and Lasco et al. [27] described an improvement in insulin resistance in two selected cohorts of postmenopausal, nondiabetic, osteoporotic women treated with Dmab, though clinically relevant effects on glucose metabolism could not be detected. The post hoc analysis of the data from the FREEDOM trial failed to show any significant effect of Dmab on fasting plasma glucose (FPG), body weight, or diabetes risk [28]. Nonetheless, in a further post hoc analysis considering diabetic patients [29], treatment with Dmab was associated with a significant reduction in FPG in antihyperglicemic drug-free diabetic women. Abe et al. [30] recently reported a reduction of HbA1c levels in a cohort of osteoporotic patients with T2DM after one year of Dmab treatment, strengthening the hypothesis that the impact of RANKL blockade may be more relevant in presence of overt abnormalities of glucose homeostasis.

Since women with breast cancer treated with AIs represent a population at higher risk of metabolic disturbances, aim of the present study was to evaluate in a cohort of postmenopausal, nondiabetic, AI-treated women with breast cancer the effect of a single 60 mg dose of denosumab on parameters of glucose metabolism, including circulating levels of hepatic-released FGF21, a metabolic regulator with multiple effects on lipid and glucose metabolism.

## 2. Patients and Methods

### 2.1. Patients

Fifteen patients were recruited from the Bone Metabolic Outpatients Clinic of ASST-Nord Milano and Istituto Ortopedico Galeazzi in Milan from June 2017 to October 2018. The study was approved by the local ethical committee, and written informed consent was obtained from each participant. Postmenopausal patients with breast cancer treated with an AIs (3 with exemestane, 7 with anastrozole, and 5 with letrozole) for less than 12 months but more than 6 months were included. Exclusion criteria were: age >80 years, regular menses or last menses from less than 6 months, pregnancy, overt diabetes mellitus, concomitant glucocorticoid or anticonvulsant treatments, concomitant conventional chemotherapy, active endocrinopathy (except well-controlled hypothyroidism), alcohol abuse, chronic kidney failure, liver diseases, malignancies other than breast cancer, and current or previous osteoporotic treatment except calcium and vitamin D supplementation. All patients were naïve to Dmab treatment and supplemented with 1000 UI/day cholecalciferol.

### 2.2. Study Design

This is a prospective observational pilot study. Patients were clinically and biochemically evaluated after overnight fasting at baseline, 1 and 5 months after a single subcutaneous administration of 60 mg Dmab. Anthropometric features, including body mass index (BMI) and waist circumference, were recorded at each visit.

At baseline, a complete evaluation of calcium-phosphate metabolism was performed, including serum calcium, phosphate, total alkaline phosphatase (ALP), albumin, parathyroid hormone (PTH), 25-OH vitamin D (25OHD), C-terminal telopeptide of type 1 collagen (CTX), and protein electrophoresis. Lipid profile was evaluated by measuring serum total and HDL cholesterol and triglycerides to exclude the presence of relevant dyslipidemia that could be a cause of insulin resistance. Furthermore, glucose metabolism was investigated by determination of serum glucose, insulin, and HbA1c levels. All participants were tested with a 75 g oral glucose tolerance test (OGTT), as previously described [26]. Baseline insulin resistance was assessed by HOMA-IR Index (HOmeostasis Model Assessment for Insulin Resistance), calculated according to the formula ((fasting insulin × fasting glucose)/22.5) [31]. Insulin response to the oral glucose load was estimated by calculating the ΔAUC (area under the curve) of insulin using the trapezoidal integration rule [32]. First-phase insulin secretion, representative of the *β* cell function, was calculated from the OGTT data by the method of the insulinogenic index, modeling the change in serum insulin divided by the change of plasma glucose from 0 to 30 min [33]. Insulin sensitivity was determined by the QUICKI Index (Quantitative Insulin Sensitivity Check Index), calculated according to the formula 1(log fasting insulin (mcU/mL) + log fasting glucose (mg/dL)) [31]. We further assessed the insulin sensitivity from OGTT data according to the surrogate marker Matsuda Index, including plasma glucose and serum insulin obtained at 0, 30, 60, 90, and 120 min after 75 g glucose load [31]. Finally, serum levels of fibroblast growth factor 21 (FGF21) were measured.

One month after Dmab injection, all patients were reassessed by a basal sample for the determination of CTX and total ALP to evaluate the acute effect of Dmab on bone turnover, and for serum glucose, insulin, FGF21, and repeating the OGTT.

Five months after Dmab injection, metabolic bone parameters as well as serum HbA1c levels were reevaluated in all participants.

### 2.3. Biochemical Assays

Serum calcium, phosphate, total ALP activity, plasma glucose, and HbA1c were routinely assayed on a chemical analyzer Architect c8000 (Abbott). Serum insulin, 25OHD, and plasma PTH were evaluated by ECLIA assays (electrochemiluminescence immunoassays) on COBAS c800 (Roche). Serum FGF21 was measured by the HUMAN FGF21 DY2539 assay kit (R&D System).

### 2.4. Statistical Analysis

Statistical analyses were performed using GraphPad Prism 7.04 Software. Data were expressed as mean ± SD. Each variable was tested for normality by D'Agostino–Pearson test. For normally distributed variables, data obtained at different time points were compared using Student's *t*-test for paired data, or one-way analysis of variance (ANOVA) in case of multiple comparisons. Nonparametric data were analyzed by the Wilcoxon signed-rank test and nonparametric Friedman test in case of multiple comparisons. Association between variables was assessed by Pearson correlation coefficient and linear regression. Sample size has been estimated by considering as significant the detection of improved QUICKI Index as significantly different mean levels between baseline and one month after Dmab; we tested the hypothesis that QUICKI improved in at least 70% of tested patients; using a binomial test with two tails, and stating a *α* error of 0.05 and a power of 0.90, the total sample size was defined of 14 patients; sample size calculation was performed by G*∗*power. A *p* value ≤0.05 was considered statistically significant.

## 3. Results

Of the 15 enrolled patients, one was excluded due to the diagnosis of previously unknown diabetes at baseline evaluation. A total of 14 patients (mean age, 68.1 ± 8.1 yrs) completed the 5-month follow-up. Clinical and biochemical data are shown in Table 1. All patients received adequate vitamin D supplementation, as indicated by serum 25OHD levels above 30 ng/ml.

### 3.1. Effects of Dmab on Bone Metabolism

Subcutaneous administration of 60 mg of Dmab was effective on bone metabolism as demonstrated by reduced CTX and ALP levels both one month (0.66 ± 0.29 versus 0.05 ± 0.03 ng/mL, *p*=0.0006 and 88.9 ± 25.2 versus 75.2 ± 18.4 U/L, *p*=0.018, respectively) and 5 months after Dmab administration (0.66 ± 0.29 versus 0.10 ± 0.11 ng/mL, *p*=0.002 and 88.9 ± 25.2 versus 58.7 ± 6.9 U/L, *p*=0.018, respectively). No significant changes in serum total calcium, phosphate, and 25OHD levels occurred, while serum PTH levels increased at 5 months compared to baseline (36.1 ± 14.1 versus 49.6 ± 14.5 pg/mL; *p*=0.008). Body weight and waist circumference did not change during the observational period. No clinical fragility fracture occurred.

### 3.2. Effects of Dmab on Glucose Metabolism

Dmab administration did not affect basal and OGTT-related glucose metabolic parameters at 1 month (Table 2). In particular, mean fasting glucose and insulin levels did not differ before and 1 month after Dmab administration. Data derived from OGTT showed that glucose levels at 60 min one month after Dmab administration were higher compared with 60 min glucose levels at baseline (166.5 ± 38.2 versus 146.3 ± 21.4 mg/dL; *p*=0.034), while glucose levels at 120 min were lower (126.2 ± 35.8 versus 142.2 ± 31.5 mg/dL; *p*=0.021). Nonetheless, insulin levels did not show differences when compared to baseline at each OGTT time point, nor did mean AUCs of insulin levels. Similarly, none of the calculated indexes was affected by Dmab (Table 2), though HOMA-IR and insulinogenic indexes showed a trend toward reduction (2.01 ± 1.52 versus 1.81 ± 1.05; *p*=0.27 and 0.78 ± 0.64 versus 0.64 ± 0.48; *p*=0.45, respectively), while Matsuda and QUICKI Indexes showed a nonsignificant trend to increase (5.79 ± 3.51 versus 7.64 ± 8.58; *p*=0.26 and 0.362 ± 0.043 versus 0.365 ± 0.042; *p*=0.51, respectively). Circulating HbA1c levels increased 5 months after Dmab injection (36.0 ± 2.3 versus 39.6 ± 3.1 mmol/mol; *p*=0.01).

**Figure 1:**
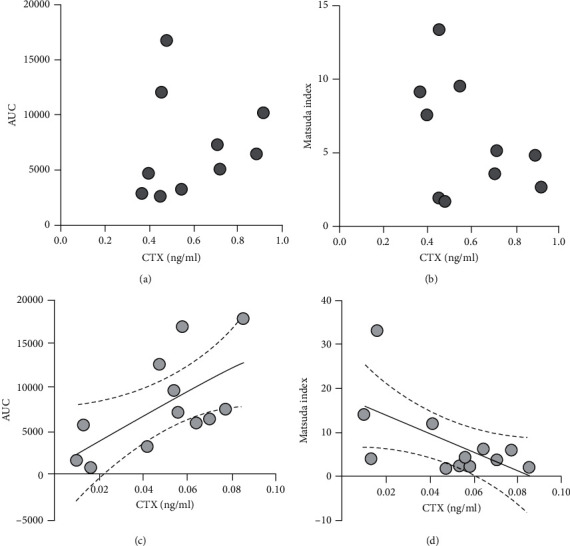
Correlations between serum CTX levels and OGTT-derived indexes of insulin sensitivity in postmenopausal breast cancer women treated with AIs at baseline and 1 month after 60 mg Dmab administration. At baseline evaluation, no significant correlation could be detected between serum CTX levels and both insulin AUC (a) and Matsuda Index (b) while 1 month after Dmab injection, suppressed CTX levels positively correlated with insulin AUC (c) and negatively with Matsuda Index (d). Continuous line, linear regression; dashed lines, 95% confidence interval. AUC, area under the curve of serum insulin levels after 75 g oral glucose load; CTX, C-terminal telopeptide of type 1 collagen.

Both at baseline and one month after Dmab administration, expected correlations between anthropometric parameters, namely, BMI and waist circumference, and insulin sensitivity were preserved. BMI correlated with HOMA-IR Index both at baseline (*r* = 0.788, *p* = 000.2) and 1 month (*r* = 0.714, *p* = 0.009) after Dmab injection; waist circumference correlated with HOMA-IR and QUICKI Index both at baseline (*r* = 0.701, *p* = 0.011 and *r* = −0.607, *p* = 0.036, respectively) and 1 month (*r* = 0.696, *p* = 0.012 and *r* = −0.612, *p* = 0.034, respectively) after Dmab injection. Of note, though basal serum CTX levels failed to show a significant correlation with any metabolic parameter, Dmab-suppressed CTX levels positively correlated with insulin AUC (*r*^2^ = 0.404, *p* = 0.026) and negatively with Matsuda Index (*r*^2^ = 0.352, *p* = 0.042) (Figure 1).

### 3.3. Effects of Dmab on Circulating FGF21 Levels

Interestingly, though in AI-treated women glucosensitivity was not changed, serum FGF21 levels were reduced 1 and 5 months after Dmab administration (138.7 ± 44.0 versus 106.8 ± 43.4 versus 73.3 ± 24.8 pg/mL; *p*=0.024 by one-way ANOVA).

### 3.4. Effects of Dmab on Glucose Metabolism in the Subgroup of Insulin-Resistant Patients

The subgroup of insulin-resistant women (*n* = 5), defined on the basis of HOMA-IR ≥ 2.5 and of Matsuda Index ≤2.5 at baseline, was considered. In the present cohort of nondiabetic postmenopausal women treated with aromatase inhibitors, HOMA-IR levels correlated with Matsuda index levels (*r* = -0.828 and *p*=0.002). In the subgroup of insulin-resistant women, a reduction in fasting insulin levels was detected one month after Dmab administration (16.3 ± 4.9 versus 13.5 ± 3.5 mcU/mL; *p*=0.029). Moreover, QUICKI Index increased (0.317 ± 0.013 versus 0.327 ± 0.009; *p*=0.025), while HOMA-IR, Matsuda Index and Insulinogenic Index did not show significant variations. Though serum FGF21 levels in insulin-resistant women did not differ from those detected in normoinsulinemic women (129.9 ± 67.4 versus 127.1 ± 19.7 pg/ml), they were reduced one month after Dmab administration (129.9 ± 67.4 versus 105.7 ± 60.2 pg/ml; *p*=0.032). Finally, at variance with what was detected in the whole series, HbA1c levels remained unchanged from baseline to the end of the study period (35.6 ± 2.3 versus 38.3 ± 2.1 mmol/mol; *p*=0.37).

## 4. Discussion

Women with breast cancer treated with aromatase inhibitors (AIs) are a population in which the coexistence of imbalance of bone and glucose homeostasis, similar to that detected in diabetic patients [34], could represent a model to elucidate the effect of denosumab (Dmab) on glucose metabolism. Therefore, we prospectively evaluated postmenopausal, nondiabetic, AI-treated women with breast cancer in basal condition and one month after a single 60 mg Dmab injection, when circulating levels of the drug reach their maximal levels [35]. We also assessed glycated haemoglobin (HbA1c) of the patients five months after Dmab administration to evaluate the midterm glycemic control.

As expected, Dmab inhibited bone resorption as documented by significant decreases of both circulating ALP and, in particular, CTX levels. Plasma PTH levels increased in the absence of a significant reduction of serum albumin-corrected calcium levels, and none of the patients experienced hypocalcemia or fragility clinical fractures. A single dose of 60 mg Dmab was neutral on BMI and waist circumferences as well as on the correlations between anthropometric parameters and indexes of insulin sensitivity.

Despite the study failed in detecting improvement of insulin-resistance indexes, HOMA-IR, and Matsuda Index as well as of insulin sensitivity index QUICKI, one month after a single 60 mg Dmab injection, FGF21 levels were significantly reduced, consistently with the improvement of hepatic insulin sensitivity, and Dmab inhibitory effect on FGF21 was detectable also 5 months later. These data are in line with those reported in postmenopausal otherwise healthy osteoporotic women [26, 27]. Fibroblast growth factor (FGF) 21 is a circulating polypeptide hormone that modulates several pathways involved in fatty acid and glucose metabolism [36]. FGF21 is secreted mainly by hepatocytes and to a lesser extent by skeletal muscle, adipocytes, and pancreatic cells [36]. Elevated circulating FGF21 levels have been associated with conditions characterized by hepatic insulin-resistance (metabolic syndrome [37], nonalcoholic fatty liver disease (NAFLD) [38], and T2DM [39]). Since RANKL modulates liver insulin sensitivity, we tested the hypothesis that this effect may involve FGF21 release from the liver. The detection of an inhibitory effect of Dmab on circulating FGF21 levels may suggest a crosstalk between RANK/RANKL system and FGF21; consistently, involvement of FGF21 in the metabolic bone-liver axis has been experimentally described in animals [40] and humans [41], where FGF21 promotes insulin sensitivity but causes bone loss, promoting a proosteoclastogenic activity mediated by insulin-like growth factor binding protein 1 (IGFBP1).

On the other hand, mean HbA1c levels of our patients increased at the end of the study, suggesting a deterioration of midterm glycemic control. This apparent discrepancy may be related to several explanations. It has been suggested that Dmab exerts a detrimental effect on insulin secretion through a reduction of circulating osteocalcin, an osteoblast derived protein that promotes the activity of pancreatic *β* cells [28]. Conversely, Kondegowda et al. showed that Dmab stimulates *β* cell proliferation through inhibition of the NF-*κ*B ligand pathway [42]. In our cohort, the insulinogenic index, exploring the defect in early insulin secretion after glucose load, decreased slightly one month after Dmab administration. Although this finding may explain the worsening in glycemic levels 60 minutes after glucose load, its clinical impact on midterm glucose control remains to be clarified. Since pharmacokinetic analyses showed that Dmab levels are highest four weeks after the injection [35], it is also possible that the positive effect of the drug on insulin sensitivity is relevant only at its peak and progressively vanishes with the decline in its circulating levels. In this regard, the improvement in HbA1c levels reported by Passeri et al. [26] could be attributable to the shorter (12 weeks) interval between Dmab injection and metabolic assessment in their protocol. Furthermore, the increase of HbA1c levels observed in our patients may reflect a progressive worsening of glucose homeostasis, already described in women treated with AIs [14], rather than a detrimental effect of Dmab.

Data from our study are in line with previous reports, showing that the impact of a single Dmab injection on glucose metabolism is unable to exert a relevant clinical effect [26–29]. Though long-term treatment with Dmab reduces the risk of vertebral fractures and increases BMD in both diabetic [43] and nondiabetic osteoporotic women [44], it remains controversial whether a prolonged blockade of the RANKL/RANK pathway can significantly affect glucose metabolism. Schwartz et al. [28] did not detect any significant effect of Dmab on metabolic parameters of osteoporotic women enrolled in the FREEDOM study, while in a further post hoc analysis of the same trial, Napoli et al. [29] found that treatment with Dmab was associated with a reduction of FPG in drug-free diabetic women. More recently, Abe et al. [30] reported a positive effect of one-year Dmab treatment on insulin resistance and HbA1c in a cohort of osteoporotic patients affected by T2DM. These observations suggest that RANKL blockade may be clinically relevant in the presence of abnormalities of glucose homeostasis, as suggested by previous animal and clinical studies [45]. Based on this hypothesis, we performed a subgroup analysis of insulin-resistant women, who displayed an improvement of both hepatic insulin resistance and fasting insulin levels one month after Dmab administration. Since high circulating RANKL levels have been associated with both insulin resistance and the risk of developing T2DM [24, 45], it is likely that Dmab exerts a significant effect in patients with insulin resistance due to a greater inhibition of the RANKL activity. This hypothesis is consistent with the finding that Dmab suppressed-CTX levels positively correlated with insulin AUC and negatively with the Matsuda Index.

Although the present study suffers from some limitations due to small sample size, short-term investigation after only one dose of Dmab, and lack of a control group, inclusion of a control group would have been unethical, since it is known that delaying Dmab therapy increases fracture risk of patients treated with AIs [22]. However, while the selection of a homogeneous population without interfering clinical and pharmacological factors prevented the enrollment of a greater number of patients, it allowed the detection of Dmab effect on glucose metabolism. Finally, though Dmab-induced changes in FGF21 levels are of interest, the sample size of the study, admittedly, was not powered to detect FGF21 changes.

In conclusion, Dmab is confirmed to be safe for glucose metabolism in both normal and insulin-resistant women with breast cancer treated with AIs. This issue is clinically relevant as AI-treated women are affected by a high prevalence of metabolic abnormalities. Our data confirm that Dmab may induce a short-term positive effect on insulin sensitivity, mainly at hepatic level, where RANKL is highly expressed. Nonetheless, this effect seems to be too small to exert a clinically protective role in the long term. Insulin-resistant patients are likely to benefit more from treatment with Dmab than normosensitive patients. Lastly, data suggest a crosstalk between RANKL and FGF21 in the modulation of glucose homeostasis.

## Figures and Tables

**Table 1:** Clinical and biochemical features of the postmenopausal breast cancer patients at baseline and 5 months after 60 mg Dmab injection.

	Baseline	5 months	*p* value
Weight (kg)	58.7 ± 13.0	57.8 ± 13.0	0.30
BMI (kg/m^2^)	23.5 ± 4.6	23.3 ± 5.0	0.39
Waist circumference (cm)	81.7 ± 12.2	77.4 ± 16.4	0.12
Serum alb-corr calcium (mg/dL)	9.7 ± 0.4	9.6 ± 0.4	0.81
Serum phosphate (mg/dL)	3.5 ± 0.4	3.3 ± 0.4	0.56
Serum 25OHD (ng/mL)	34.3 ± 11.3	33.8 ± 11.6	0.90
Plasma PTH (pg/mL)	36.1 ± 14.1	49.6 ± 14.6	**0.008**
Total ALP (U/L)	88.9 ± 25.2	58.7 ± 6.9	**0.045**
Serum CTX (ng/mL)	0.7 ± 0.3	0.1 ± 0.1	**0.002**
HbA1c (mmol/mol)	36.0 ± 2.3	39.6 ± 3.1	**0.01**

BMI, body mass index; alb-corr, albumin-corrected; 25OHD, 25-hydroxyvitamin D; PTH, parathormone; ALP, alkaline phosphatase; CTX, C-terminal telopeptide of type 1 collagen; HbA1c, glycated haemoglobin.

**Table 2:** Parameters of glucose metabolism of the patients at baseline and one month after denosumab injection. Data are expressed as mean ± SD.

	Baseline	1 month	*p* value
*Whole study population (n* *=* *14)*			
Serum glucose (mg/dl)	87.0 ± 8.7	83.8 ± 11.1	0.17
Serum insulin (*μ*U/ml)	9.1 ± 6.4	8.5 ± 4.8	0.31
Log ΔAUC insulin (*μ*U/ml/120 min)	7692 ± 4698	7795 ± 5244	0.90
HOMA-IR Index	2.0 ± 1.5	1.8 ± 1.1	0.27
Matsuda Index	5.8 ± 3.5	7.6 ± 8.6	0.26
QUICKI Index	0.362 ± 0.043	0.365 ± 0.042	0.51
Insulinogenic Index	0.8 ± 0.6	0.6 ± 0.5	0.45

*Patients with HOMA-IR >2.5 (n* *=* *5)*			
Serum glucose (mg/dl)	90.6 ± 5.3	87.2 ± 4.9	0.44
Serum insulin (*μ*U/ml)	16.3 ± 4.9	13.5 ± 3.5	**0.029**
Log ΔAUC insulin (*μ*U/ml/120 min)	12429 ± 3883	12745 ± 4581	0.63
HOMA-IR Index	3.69 ± 1.29	2.89 ± 0.61	0.09
Matsuda Index	2.46 ± 0.71	2.65 ± 0.96	0.54
QUICKI Index	0.317 ± 0.013	0.327 ± 0.009	**0.025**
Insulinogenic Index	1.12 ± 0.95	0.82 ± 0.65	0.46

ΔAUC insulin, variation of area under the curve of insulin; HOMA-IR, homeostasis assessment model of insulin resistance; QUICKI, Quantitative Insulin Sensitivity Check Index.

## Data Availability

The data used to support the findings of this study are available from the corresponding author upon request.
